# Tape-Worm

**Published:** 1872-04

**Authors:** R. C. Word

**Affiliations:** Tunnel Hill, Ga.


### TAPE-WORM.

BY R. C. WORD, M. D., TUNNEL HILL, GA.

In the *New Orleans Journal of Medicine*, Jan. No., 1868, we published an article on the subject of Tape Worm in the human subject. We have been asked to republish that article in the COMPANION, but as its length would not comport with the motto, "Quicquid Præcipies," etc., we will submit only an abridgement of the paper referred to.

During the years 1865 and 1866 no less than ten cases of Tænia were reported to the Atlanta Medical Society. Two of these were reported by the writer, who has since treated two—although in a practice of twenty years previous to the war, he had not treated a single case. The cause of the extraordinary frequency of tape worm since the late war, is an interesting subject of inquiry.

The subject of parasites in general is one of singular interest to the student of natural history; and the attention of the Pathologist and physician is now called to the development of new and important facts connected with the origin and propagation of parasites in the human system.

Every animal has its peculiar parasites, and even the parasites themselves have parasites. Said Hudibras,

> "These fleas have other fleas to bite 'em,
> And these fleas, fleas, ad infinitum."

About thirty forms of entozoa have been discovered occupying the human body. Until recently, only two varieties of the cestoid family, or tænia, were known to infest the human subject, to wit: the *Tænia Solium* and *Tænia Lata.* Now no less than eight varieties are recognized, and two varieties of the bothriscephalus.

The *Tænia Solium* is the most common in the United States, and is the variety now prevalent in the South. The *Tænia Mediocanellata,* said to be frequent in Europe and South Africa, probably exists here, but is confounded with the *Tænia Solium.*

For a classification of the genera and species of these entozoa, we refer the reader to Aitkin's late work on Practice.

Each of the several varieties of Tænia must first exist as a cysticercus, larva, or non-sexual embryo, before it can become a fully developed tape worm.

It was not known, until a comparatively recent date, that the hydatid, or viscicular worms, found in the flesh of various animals, are the young or larvæ of the tape worms. These larvæ or cyst worms, technically termed *cysticerci,* are the second stage of the tape worm, and consist of a tænia head united by a neck to a vesicular body. The cyst which envelopes the head and neck is developed at the expense of the adjacent tissue. It is shaped like a bladder, the head of the worm corresponding to the neck of the bladder, and is usually retracted within the body, like the neck of an inflated bladder drawn within itself. The cysts are frequently as large as an English pea. As these parasites have no generative organs, they have been long known as sexless entozoa, and at one time were accounted for on the theory of spontaneous generation, a doctrine which has few if any advocates at the present day—the theory of "Omne vivum ex ovo" being now generally accepted.

The relation which these encysted entozoa hold to the tape worm is the same as that of the caterpillar to the butterfly. The process of fecundation, and the development of the embryo from the ovum, have now been actually observed in a considerable number of the parasitic entozoa; and it is to be remembered as a general fact, that the development of the ova rarely takes place in the same animal, or in the same part of an animal, in which the parent worm has passed its life, and has exercised the generative functions. There is a migration from a parasitic to a free condition for a time (e. g. *Ginuea-worm*, *Ascarides*); or from one animal into another animal, the free condition intervening (e. g. Bothriocephalus): or, lastly, the migration may take place from one part to another of the same animal (e. g. *T. Spiralis*) (*Aitkin*).

The Tænia Solium, so frequently found in the human intestine, is doubtless in most instances derived from the cysticercus cellulosa of the hog. The innumerable ova deposited by the tape worm, and which come away by thousands in the segments that are discharged from the bowels, when eaten by the hog are not immediately hatched into tænia in the bowels of the animal, but must first find entrance, in some way, into the solid tissues, where it becomes a cysticercus as above described. So long as it remains encysted in the flesh of the animal there is no development beyond the embryo state. It is simply a larva, or semi-developed tape worm, and requires for its matu ity, that it be taken into the stomach of man, thence into the small intestines, where it finds its proper habitat; and seizing upon the mucous membrane, it immediately enters upon its growth toward a full formed tænia. As the caterpillar, hatching from the egg of a butterfly, enters the crysalis state to become a butterfly, so the cysticercus, hatching from the egg of a tape worm, must enter the small intestines of man to become a fully developed tape worm.

The law which governs the development of tænia was first promulgated by Steenstrup, under the name of *alternating generations*, from which the doctrine was deduced that the whole of the cystic family of entozoa are nothing else than the larvæ of the cestoid or tape worm family. The relation between the animals infested with tænia, and those containing the cyst worm seems to be that of devourer and prey. Thus the cysticercus facioloris is a cyst worm peculiar to the mouse and rat, while the cat, the natural devourer of these animals, is subject to the tænia crassicollis, the head of which so strikingly resembles the head of the cysticercus fasciolaris as to leave no doubt of their identity. So the cysticercus piseformis of the hare answers to the tænia crassiceps of the fox—there being a like resemblance between the heads of the two. And so the cysticercus cellulosæ found in the ox, sheep, goat, etc.—and especially in the hog—has been shown to be the embryo of the tænia solium, common to the human subject.

Kucheinmeister, a German naturalist, fed dogs and cats with flesh containing cyst worms, and afterwards found in their intestines tape worms corresponding to them. He also contrived to mix cysticerci collected from pigs and rabbits, with the food of a criminal under sentence of death. These were swallowed at various periods from seventy-two to twelve hours before his decapitation. Forty-eight hours after it, ten young tape worms were seen attached, by their hooks and suckers, to his small intestines.—(Watson.)

Whether the several cysts and tape worms are distinct in species, or whether the varieties may not result from the animals they feed upon, has not as yet been clearly ascertained. So far as the evidence goes, however, it appears probable that, in most instances, the animal in which the final development occurs, determines the variety.

The small intestines appear to be the natural habitat for tænia in the human system. Here they become sexually mature, and deposit their ova. Of these, one in a million perhaps, when discharged with the fæces, and in the joints that pass from time to time, finds entrance in some fortuitous way into the body of some animal, where, becoming metamorphosed into a cysticercus, it awaits the chances, rare and uncertain, of finding entrance into the small intestines of man, for its final growth and development.

The habit of the hog in eating fæcal matter, and filth of every kind, readily accounts for his great liability to cysticerci. The ova, being taken into his mouth and stomach, find entrance through the circulation and otherwise into the solid parts of his body.

The disease known as measles with hogs is caused by the cysticercus cellulosa. Each vescicle which spots the flesh of the animal, in this disease, contains one of these cyst-worms. In this we find a ready solution of the extraordinary frequency of tape worm during the late war. It is known that a great deal of raw bacon was consumed by our soldiers during the war, and that not unfrequently the home people, particularly among the poorer classes, partook of raw meat in the rations furnished by the Government. Large quantities of measly pork were slaughtered, and in the hurry and recklessness of commissary agents, packed and distributed to the army.

As the cysticerci also infest the flesh of the ox, raw beef may be regarded as a not infrequent source of tænia. The cysticerci are tenacious of life, and are probably not destroyed by a less degree of heat than 212°. So not only the chipped beef, but the rare beef of our fashionable hotels are hazardous articles of diet. In Abyssinia—where the tape worm is more prevalent than chills and fever in this country—the inhabitants are addicted to the use of raw meat. Parkins, in his work called "Life in Abyssinia," thus remarks: "In speaking of the diseases of the country, I would begin with the most prevalent. *Tœnia*, or tape worm, is, on this account, certainly the first to be concidered; for the whole population may be said to be affected with it." He mentions the fact that many attribute the disease to the eating of "broundo," or raw beef. Brice, another Abyssinian traveller, in alluding to the slaughtering of animals in that country, states that "Almost before the death struggle is over, persons are ready to flay the carcass, and pieces of raw meat are cut off and served up, before the operation is complete; in fact it is cut off and eaten while yet warm and quivering."

The recent discoveries connected with the development of the cestoid larvæ have awakened much interest on the subject, especially in Germany. It is probable that deductions drawn from analogies in the development of insect tribes, are destined to give aid and impetus to investigations on this subject. The object of the gad-fly in depositing her eggs upon the hair of horses, and the instinctive aversion of the animal to the presence of the insect, has been ever regarded as singular and mysterious; though long supposed in some way connected with the worm known as *the bott*; yet the manner of its introduction to the intestines of the animal being unknown, few people were inclined to accept a theory apparently so unreasonable and impracticable.

It is now known, however, that the bott is the larvæ of the gadfly; for you have only to put one of these "nits," or eggs, upon the tongue of the horse, and in one minute's time it will hatch into a crawling larva under the eye of the observer. If the eggs be put into a little saliva in the palm of the hand, and the breath blown warmly upon them for a few moments, the same result will follow, as the writer has himself observed.

It is highly probable that other parasites than tænia enter the human system in some manner analagous to the above.

We have not space to describe the tænia found in the human subject. The best description we have seen of the Tænia Solium is that given by Prof. Watson. We have treated seven cases of tape worm, five of which were successful and two doubtful. A case is not regarded as certainly relieved unless the head is found. We have retained two specimens of the worm—one of which is fifteen feet, the other eight feet long; and have full notes of the above cases, which we may publish at a future time.

In regard to the symptoms of tape worm, it may be remarked, that in children, and in persons of delicate and nervous temperaments they are usually well marked and distressing; yet it is questionable whether they are worse than would be occasioned by the presence of an undue number of lumbricoids or other variety of intestinal worms, in the same subject.

Among the several remedies recommended by Authors for the removal of tænia, the following are regarded as most efficacious: The *etherial oleo-resinous extract of the felix mas*, or the *male shield fern*, in doses of twenty to twenty-four grains. The *Kameela* of the natural order *euphorbiaceæ*, and sub-order *cratoneæ*. One to three drachms of the powder is the dose, given in mucilage or syrup. Lastly, the *Kooso*, or *Cusso*. The *Kooso* is an Abysynian ornamental tree, and was first brought to the notice of the Profession by Dr. Brayer, of Constantinople, in the year 1823, in honor of whom its botanical name, *brayera anthilmentica*, was first adopted. We regard the *Kooso* properly administered, as infallable for the removal of tape worm. It is well to remark, that the loose flowers contained in the shops are often inert from age and exposure. The preparation we use is the pulverized Kooso prepared by Grimault & Co., Philadelphia. It is the flowers of the tree powdered when fresh, and tightly corked in bottles containing single adult doses of half an ounce, which is directed to be given in infusion on an empty stomach. It operates in two to four hours, bringing away the worm. If it fails to operate, a purgative of castor oil and turpentine should be given.

